# Factors Associated with Unmet Needs among African-American Dementia Care Providers

**DOI:** 10.4172/2167-7182.1000267

**Published:** 2016-01-22

**Authors:** PJ Desin, AM Caban-Holt, EL Abner, LJ Van Eldik, FA Schmitt

**Affiliations:** 1 Sanders-Brown Center on Aging, University of Kentucky, Lexington, Kentucky, USA; 2 Graduate Center for Gerontology, University of Kentucky, Lexington, Kentucky, USA; 3 Department of Epidemiology, University of Kentucky, Lexington, Kentucky, USA; 4 Department of Anatomy and Neurobiology, University of Kentucky, Lexington, Kentucky, USA; 5 Department of Neurology, University of Kentucky, Lexington, Kentucky, USA; 6 Department of Behavioral Science, University of Kentucky, Lexington, Kentucky, USA

**Keywords:** Dementia, Caregiving, Family, Minority aging, Socioeconomic status, Services, Needs

## Abstract

Racial and ethnic minorities currently comprise 20% of the U.S. population; in 2050, this figure is expected to rise to 42%. As a result, Alzheimer’s disease (AD), the 5^th^ leading cause of death for people aged 65 and older, is likely to increase in these groups. Most dementia caregiving for these populations comes from family and friends, especially among families with lower socioeconomic status. A convenience sample of 30 African-American dementia caregivers was interviewed to determine unmet needs. Participants expressed a limited desire for formal services, such as support groups, legal advice, case management, and homemaker services. Instead, commonly expressed needs were daytime respite care and especially a desire for family and social support. Many caregivers expressed a need for other family members to share responsibility in the process; therefore, methods for caregiver support that address multiple family members in care provision may be beneficial for this group.

## Introduction

Dementia defines a clinical syndrome with progressive loss of cognition and functional ability [1]. Alzheimer’s disease is the 5^th^ leading cause of death for individuals over age 65 in America and is the most common form of dementia, afflicting an estimated 5.3 million older Americans. According to Borson et al. [2] the number of individuals with dementia is expected to double by 2050. Racial and ethnic minorities comprised 20% of the population as of 2014, but are expected to reflect 42% of the population by 2050 [3] and are likely to experience an increasing dementia care burden. Informal caregiving by friends and family is the backbone of America’s long term healthcare system, as approximately 65% of the older population with long term care needs depend on family and friends for assistance [4]. Over 15.4 million informal caregivers are currently providing unpaid assistance to persons with AD or other forms of dementia [5,6]. Informal caregiving for dementia, currently at 60–80% of all dementia care, is expected to increase proportionally as the number of persons diagnosed with dementia increases [1].

African-American caregivers may experience a disproportionate care burden because of their minority and/or lower socioeconomic status. Non-white populations of family care providers have been described as more likely to be an adult female offspring who is younger, with children of her own under 18 years of age, economically disadvantaged, with less education, often unemployed, and with more health issues than her white counterpart [3]. Further, African-American caregivers for persons with dementia (PWD) spend more time and experience a higher burden from caregiving than non-Hispanic whites [5]. Given these findings, it is important to focus on the perceptions of African-American caregivers regarding their provision of assistance to PWD, their unmet needs while caregiving, and the implications of these needs for tailoring supportive services for this group. AD is considered a “silent epidemic” in the African-American community, due to lack of public awareness of the scope of the problem related to socioeconomic status and other risk factors, despite its relatively high incidence and significant contribution to increased mortality [7]. The reluctance to acknowledge a potential memory problem outside of the family for fear of embarrassment and disrespect for the individual’s status in the community contributes to the late stage initial diagnosis often seen in African-American dementia patients, which significantly reduces the already limited potential benefits that can be rendered by currently available treatments [8]. For these reasons, it is important to examine the potential impact of caregiving on African-American dementia caregivers. The purpose of the current study is to describe the unmet caregiving needs of 30 African-American dementia caregivers in a midsized Southeastern metropolitan area.

## Materials and Methods

### Participants

The study was conducted at the University of Kentucky by investigators at the Sanders-Brown Center on Aging Alzheimer’s Disease Center (ADC). The African-American caregivers in this investigation were a convenience sample identified by a minority outreach coordinator working with the African-American Dementia Outreach Partnership [9] and were initially contacted by telephone to determine their willingness to participate in the study. Out of a total of 32 caregivers contacted, 30 agreed to participate. In-person interviews were carried out by one of the authors (AC-H) in caregivers’ homes, and a reimbursement of 50 dollars was offered to compensate for their time. Each caregiver provided demographic data and completed a structured interview about their caregiving experiences. All research activities were approved by the University of Kentucky Institutional Review Board, and each participant provided written informed consent.

### Measures

#### Community care demographics

This three-item questionnaire collected data on background characteristics of the caregiver, care recipient’s living status, caregiver subjective well-being, caregiving onset, and range of care provided to the person with dementia [10].

#### Activities of daily living (ADL) scale and instrumental activities of daily living (IADL) scale

These scales sought to measure caregiver reported care demands and included the extent of disability exhibited by the care recipient on nine Activities of Daily Living (ADL) tasks and eight Instrumental Activities of Daily Living (IADL) tasks. The inter-rater reliability is 0.986 and test-retest is excellent, per Sheikh, et al. [11] The caregiver responses were recorded as needs “No help,” “Some help,” or “A lot of help” [12]. For analysis, presentation categories were collapsed into “No help” vs. “At least some help.”

#### Unmet need items

This questionnaire appraised caregivers’ unmet needs. Caregivers responded to items regarding their perception of unmet caregiving needs in ADL, IADL, memory and behavior, times during the day when assistance was needed by the caregiver (day, night, weekend), the need for formal services, and need for social support (family, friends, church etc.). The responses were recorded in the form of “Yes” and “No” [13].

### Statistical analysis

Descriptive statistics were used to summarize caregiver and care recipient characteristics. Given the small sample size, no inferential statistics were used. All analyses were conducted using SAS 9.4^®^ (SAS Institute, Inc; Cary, NC).

## Results

### Caregiver characteristics

The average caregiver in this sample was 56 years old and female (83.3%). Most often the caregiver was the child of the care recipient and was retired (46.7%), although a relatively large proportion of the caregivers were employed full-time (36.7%). Among the caregivers, 40% were married and 36.7% had attended some college (Table 1).

### Care recipient characteristics

The average age of the care recipient was 77.56 years and 73.3% of the sample was female (Table 2). Most of the care recipients (86.7%, n=26) had been diagnosed with AD, 6.7% (n=2) with vascular dementia, and 3.3% (n=1) with frontotemporal dementia. In one case the caregiver was uncertain as to dementia diagnosis of the care recipient. The most frequently reported ADL impairments by caregivers were “taking medications” (96.7%) and “managing finances” (93.3%), with “housekeeping”, “doing laundry”, and “transportation” each being endorsed by 90% of caregivers (Table 2).

### Caregiver unmet needs

Unmet need, in the current investigation, is defined as the lack of resources or an expressed need for additional support by caregivers in dementia-specific domains such as ADL and IADL tasks, dementia symptomatology, and formal and informal sources of support. The most prevalent unmet needs found in the literature are for social support [14,15], and oversight and supervision over ADLs [16–18] especially within the African-American community [19].

Our current research reveals somewhat different caregiver needs. Our caregivers most often reported wanting more assistance with “daytime care” (40.0%), “dealing with loved one’s memory loss” (36.7%), and “managing behavior” (33.3%). Half of caregivers who wanted assistance with daytime care were currently employed either full (n=5) or parttime (n=1). Perceived need for daytime respite care was not associated with income; 75% of those who wanted help reported income of at least $25,000, while 60% of those who did not want help reported income of at least $25,000. In addition, perception of need was not associated with marital status, where half of each group were married; educational attainment, where 75% of those who needed help had more than a high school education compared to 40% of those who did not; or duration of illness, where care recipients of those who needed daytime respite care had carried a diagnosis for an average of 5.5 years, compared to 3.2 years in the group not requiring daytime respite care.

Caregivers least often indicated wanting more assistance with “nighttime care”. The most reported need for formal services was for a support group (46.7%), legal advice (40.0%), case management (e.g., someone to help connect caregivers to the right services) (36.7%), and homemaker services (36.7%) all being commonly indicated. The least requested formal services were meal delivery and counseling, which were both endorsed by only 26.7% of the caregivers. With regard to social support, the greatest need expressed was for family support (63.3%), with visits from family/friends also being highly endorsed at 56.7%. The least reported social support need was for counseling from clergy (16.7%), which could indicate either high levels of support from clergy were already provided or a lack of desire by caregivers for clergy support (Table 3).

## Discussion

Our findings present different results of caregiver needs in this African-American sample when compared to the current literature. One study by Hughes et al. [6] found the most unmet needs for caregivers of PWD to be availability of community resources (79.8%), education about dementia (64.3%), emotional (34.9%) and respite (31.3%) supports, available counseling/skill building for the caregiver role (24.8%), and special medical care provision (16.5%). Nearly 90% of all participants in the Hughes et al. [6] study expressed at least one need that was left unmet by the current system. Friedemann et al. [20] found that availability of resources was influenced by language barriers, resistance to use services due to cultural or emotional reasons, lack of eligibility, mistrust of services, or inability to afford them. Formal services are rarely used in minority populations despite the expression of greater need for formal support services than non-minority caregivers [8], perhaps due to strong preferences for home care for family members [21]. This is a problematic situation because formal service use has been linked to a decrease in perceptions of unmet needs and caregiver burden [22]. However, some studies have concluded that extended family is not necessarily an aid but may actually be a form of disappointment that adds to burden, suggesting that the mere presence of extended family may not actually extend the level of support for African-American caregivers as suggested in prior research [23,24]. Finally, results of one study [25] suggest both the care recipient and provider need emotional support. Discrepancies between the current study and previous research may be due to different study designs, populations, and data collection methods

Caregivers in our sample were most likely to be middle-aged women caring for a parent with AD. This is a consistent pattern for African Americans in current dementia caregiving research, with one Gallup survey finding 72% of mid-life caregivers caring for a parent, step-parent, mother-in-law, or father-in-law [26]. This may have other implications for this group as more than half of African-American caregivers are “sandwiched” between caring for an older person and a younger person under the age of 18 or more than one older person [5]. Many caregivers endorsed needing daytime respite care, but desire for this service was not considerably influenced by employment status, marital status, socioeconomic status, or duration of care. We note, however, that we had low statistical power to detect differences given the sample size. Caregivers appeared to manage weekend and nighttime care with less difficulty, which may reflect the relatively functionally intact nature of the care recipients. It was clear in responses from the social support ratings that caregivers were reporting a need to feel supported by family and to have social contact through visits from family/friends. All other forms of social support were much less needed. As reflected in the results, the need for counseling by clergy and support from church were not highly endorsed, although many caregivers reported that their religious faith was what helped them through caregiving difficulties. Many caregivers expressed that they felt well-supported by their church community (data not shown), and thus did not require more support from clergy. It appears being able to share experiences with others who are engaged in similar situations was of higher value than sharing experiences individually with a counselor. Whether the term “counselor” holds some stigma in this group of caregivers, and thus was not endorsed in favor of group support, is a question to be addressed in future research.

In contrast, the majority of caregivers reported that their families did not provide the level of desired support. Even when other types of support were available, especially formal support, caregivers preferred that family members share the work. In essence, they wanted family members to take more responsibility and help with caregiving rather than church members or formal support services. Lack of family member participation in caregiving appeared to be a great frustration for caregivers.

When necessary interventions are not available, unmet support needs and demands on the caregiver increase [16]. When limits of care providers are reached emotionally, physically, psychologically, and socially, the caregivers can suffer health problems of their own. In a survey of 221 caregivers, Thompson and Roger [1] found 35% of care providers had experienced a drop in quality of life since taking on the care role due to stress, feelings of helplessness and depression from inability to provide adequate care, and feeling tired from demands of the role. Efforts focused on tailoring services to more appropriately meet the unmet needs of African-American caregivers must carefully consider these issues rather than presume usefulness of services and caregiver accessibility to them. Identifying methods for meeting the need for services may benefit caregivers and increase their satisfaction with their caregiving responsibilities. Providers of care may not need more formalized or community-based services, as other studies suggest. Our research supports the need for respite care through family involvement and defining family roles in the caregiving process.

This study has some limitations, as our research group was a small convenience sample of individuals who were in some way familiar with the Sanders-Brown Center on Aging. In part, by virtue of willingness to participate in a research investigation, the individuals in the sample may not represent the general population of African-American caregivers of PWD, either in this region or nationally, and may have better access to information, resources, and services. Additionally, the interview for six caregivers took place after their care recipient had passed away. It is possible that the individuals who provided retrospective accounts of caregiving may have had a different perspective of their caregiving experience than those who were currently providing care.

Given the strongly expressed desire from caregivers that family members (not formal services) help provide care, a major consideration in supporting caregivers is to look at methods for directly addressing these needs and supporting the entire family in caring for a PWD, in an effort to alleviate stress and increase the caregiver’s sense of equity of responsibility for the caregiving burden. Perhaps this would be in the form of a group session for the family to discuss these issues and define roles, or may be as basic, for example, as providing transportation for the other family members. At the very least, educational and other supports need to be available to, and attainable by, all care providers, especially minority caregivers. Family caregivers are an invaluable resource for maintaining the health of PWD, and African-American caregivers and other minority populations are likely to face additional burdens from lack of awareness on the part of professional providers for their specific needs. Finding avenues to support them in their caregiving roles is vital for the upkeep of the healthcare system and will be increasingly critical in the coming years as the population continues to age.

As shown here, confounds to other studies suggest caregivers do not need more services as suggested in the literature, and they do have access to services if they are needed. Our interviews determined the caregivers want more family involvement and more daytime respite care. Future research could include studies of larger random samples, with a family composition or socioeconomic status focus.

## Figures and Tables

**Table 1:** Caregiver Demographic Characteristics (N=30).

Characteristic	Summary
Age, mean years ± std. dev	56.0 ± 10.6
Female sex (%)	83.3
Number of children (%)	
3–4	10.0
5–6	83.3
≥ 7	6.7
Number of children/grandchildren living with caregiver (%)	
1–2	72.4
≥ 3	27.6
Number of children/grandchildren for whom caregiver provides care (%)	
0	66.7
1–2	20.0
3–4	13.3
Education (%)	
Less than High School	16.6
High School	20.0
Some College	36.7
College graduate	26.7
Income (%)	
< $15,000	20.0
$15,000–$29,999	36.7
$30,000–$39,999	20.0
≥ $40,000	23.3
Married (%)	40.0
Employment status (%)	
Retired	46.7
Full-time	36.7
Part-time	3.3
Other	13.3

**Table 2:** Care recipient patient characteristics (N=30).

Characteristic	Summary
Age, years	77.56 ± 7.85
Female sex (%)	73.3
Living situation (%)	
Alone	20.0
With caregiver	46.7
With other relatives	6.7
In a facility	26.7
Relationship to caregiver (%)	
Parent	60.0
Spouse/partner	16.7
Sibling	10.0
Other	13.3
Deceased (%)	20.0
Diagnosis (%)	
Alzheimer’s disease	86.7
Vascular dementia	6.7
Frontotemporal dementia	3.3
Unknown	3.3
Mini-Mental State Exam *, mean ± std. dev	12.7 ± 8.9
Duration of caregiving**, mean years ± std. dev	4.3 ± 4.3
Activities of Daily Living Impairments*** (%)	
Bathing	73.3
Dressing	70.0
Toileting	46.7
Eating	13.3
Ambulating	50.0
Getting in/out of bed	33.3
Taking medications	96.7
Preparing food	80.0
Housekeeping	90.0
Doing laundry	90.0
Transportation	90.0
Managing Finances	93.3

* available for 23/30;

** available for 25/30;

*** figures represent percent of care recipients with at least some impairment.

**Table 3:** Unmet Caregiver Needs (N=30).

Caregiver need	Summary (%)
Need more assistance with	
Managing behavior	33.3
Dealing with loved one’s memory loss	36.7
Nighttime care	16.7
Daytime care	40.0
Weekend care	30.0
Need formal support	
Case management	36.7
Homemaker	36.7
Meal delivery	26.7
Support group	46.7
Caregiver training	30.0
Counseling	26.7
Financial advice	33.3
Legal advice	40.0
Need social support	
Counseling from pastor/priest	16.7
Support from church	20.0
Visits from family/friends	56.7
Family support	63.3
Feeling close to those around me	26.7
Feeling close to care recipient	30.0
